# Enhanced antiviral and antifibrotic effects of short hairpin RNAs targeting HBV and TGF-β in HBV-persistent mice

**DOI:** 10.1038/s41598-017-04170-1

**Published:** 2017-06-20

**Authors:** Lei Ye, Fangming Kan, Tao Yan, Jiaqi Cao, Leiliang Zhang, Zhijian Wu, Wuping Li

**Affiliations:** 1 MOH Key Laboratory of Systems Biology of Pathogens, Institute of Pathogen Biology, Chinese Academy of Medical Sciences & Peking Union Medical College, Beijing, 100730 China; 2 Ocular Gene Therapy Core, National Eye Institute, NIH, Bethesda, Maryland 20892 USA

## Abstract

The hepatitis B virus (HBV) causes acute and chronic liver infection, which may lead to liver cirrhosis and hepatocellular carcinoma. Current treatments including interferons and nucleotide analogs, have limited therapeutic effects, underscoring the need to identify effective therapeutic options to inhibit HBV replication and prevent complications. Previous animal models mimicking chronic HBV infection do not faithfully reflect disease progression in humans. Here, we used our established HBV-persistent mouse line with liver fibrosis to evaluate the efficacy of novel therapies. The combination of two short hairpin RNAs (dual-shRNA) against different coding regions of HBV delivered by a self-complementary AAV vector showed better antiviral effects than single shRNA both *in vitro* and in HBV-persistent mice. The dual-shRNA also exhibited stronger antifibrotic activity *in vivo*. Vector carrying shRNA against TGF-β, though did not inhibit HBV replication alone, enhanced the antiviral and antifibrotic activities of single and dual HBV shRNAs. Co-administration of TGF-β shRNA and HBV dual-shRNA decreased HBV DNA, HBV RNA, HBsAg, HBeAg, and liver fibrosis markers in serum and tissues, and improved liver morphology more effectively than single treatments. Our results suggest that the combination of shRNAs against HBV and TGF-β could be developed into a viable treatment for human HBV infection.

## Introduction

The hepatitis B virus (HBV) is an enveloped, partially double-stranded and hepatotropic DNA virus that replicates by reverse transcription. The virus can cause acute or chronic infection in the human liver, which is a global health problem. To date, approximately two billion people worldwide have been infected with HBV. Although the majority of infected individuals are asymptomatic and show complete recovery, approximately 400 million cases progress to chronic infection^1–3^. These cases show a high risk of developing liver cirrhosis and hepatocellular carcinoma (HCC)^4^. No reliable treatment is currently available for the diseases associated with HBV infection.

Current treatment for HBV infection mainly involves the use of immunomodulatory agents including interferon-α (IFN-α), pegylated IFN, and nucleoside/nucleotide analogs. IFN exerts its antiviral effect by activating antiviral enzymes or inducing an exaggerated cellular immune response against virus-infected hepatocytes. However, only approximately 10% of cases achieve a sustained response with IFN treatment^5^. In addition, IFN treatment is associated with side effects, including flu-like symptoms, fatigue, and low blood counts. Nucleoside/nucleotide analogs, which act mainly through direct suppression of viral replication, are slightly more effective than IFN, although their prolonged use is associated with drug resistance and virus reactivation following treatment withdrawal^6,7^. Novel therapeutic options against HBV infection with improved and long-lasting anti-HBV activity, reduced side effects, and the ability to prevent cirrhosis and HCC are in urgent need.

RNA interference (RNAi) is a powerful tool for the post-transcriptional regulation of mammalian gene expression by sequence-directed degradation of specific RNAs. In the treatment of HBV infection, synthetic small interference RNAs (siRNAs)^8–12^ are used to inhibit HBV replication and prevent the induction of HBV gene expression. Different chemical modifications have been introduced into synthetic sequences to increase the resistance of siRNAs against degradation and reduce nonspecific innate immune stimulation^13–15^. In addition, siRNAs are formulated in different delivery vehicles. One such anti-HBV siRNA candidate, ARC-520, is being tested in clinical trials. The results of Phase 2a trials demonstrated that it was well-tolerated and effective at reducing HBsAg in the serum in a dose-dependent manner for up to 57 days in chronic hepatitis B (CHB) patients^16^. This was the first demonstration of the direct antiviral effect of RNAi in humans, as indicated by a reduction in serum HBsAg levels^16^. However, repeated administration of siRNA to patients is needed for sustained HBV inhibition. Therefore, delivery of short hairpin RNAs (shRNAs) by adeno-associated viral (AAV) vectors, which achieve persistent shRNA expression in hepatocytes, is the preferred method. A number of studies tested AAV-delivered shRNAs for the treatment of HBV replication and showed promising results^17–19^. In most of these studies, the animal models of human chronic HBV infection did not faithfully mimic the progression of disease in patients, which hindered the accurate evaluation of treatment efficacy. A critical issue in these studies is whether inhibition of HBV by the new therapy is sufficient to prevent liver fibrosis, which leads to the development of cirrhosis and HCC. However, HBV-persistent mouse models with features of liver fibrosis are lacking.

Recently, we developed a HBV-persistent mouse line by AAV type8 (AAV8)-mediated hepatic delivery of the HBV genome^20^. This mouse line exhibited features of chronic HBV replication, including viral antigen production and viremia for up to 6 months with concomitant liver fibrosis, recapitulating the events that occur in humans during long-term HBV infection. In the present study, we used this model to evaluate the efficacy of our newly designed AAV-delivered shRNAs for the inhibition of HBV and prevention of liver fibrosis. Our major goal was to test whether AAV-mediated HBV shRNA delivery could persistently inhibit HBV replication and concomitantly prevent liver fibrosis. As TGF-β is one of the most important cytokines with regulatory functions in immune system against HBV infection, and in the process of liver fibrogenesis which could activate collagen-producing cells^21–24^, we tested whether the use of shRNA against TGF-β could enhance the antiviral and antifibrotic effects of shRNAs against HBV when used in combination.

## Results

### *In vitro* anti-HBV effect of shRNAs

**Figure 1:**
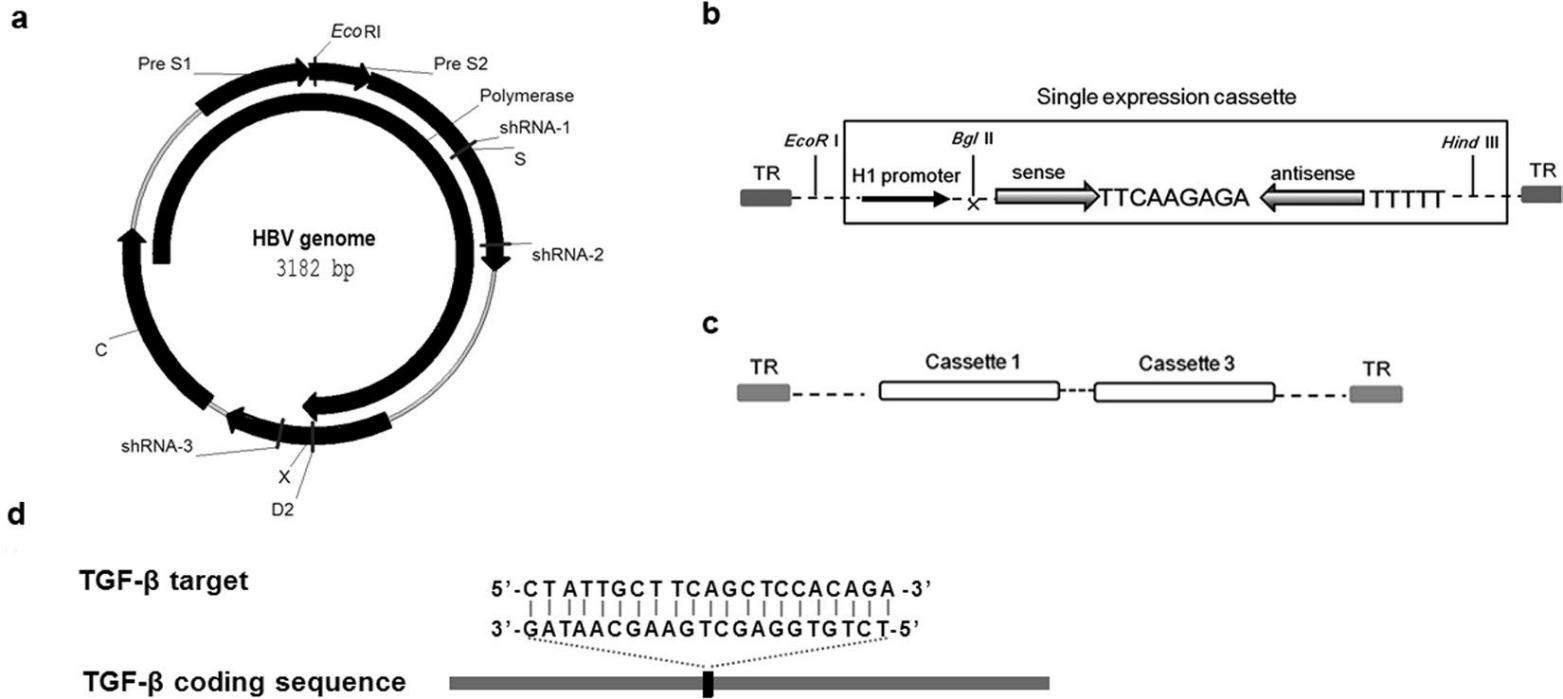
Design of shRNAs targeting HBV and TGF-β specific sites and schematic diagram of the AAV expression cassette. (**a**) Schematic of the HBV genome and the locations of the shRNA targets. (**b**) Schematic of an AAV vector containing a single shRNA expression cassette. (**c**) Schematic of AAV vectors containing dual shRNA expression cassettes. (**d**) Schematic representation of the location of the shRNA target within the TGF-β coding sequence. TR, terminal repeat.

Three shRNAs (shRNA-1, shRNA-2, and shRNA-3) targeting different coding regions of HBV (Fig. 1a) were designed using the online software siRNA Target Finder (www.ambion.com). Each shRNA expression cassette was driven by the H1 promoter and packaged into a self-complementary AAV type 2 (AAV2) vector. AAV2 vectors are used for shRNA delivery mainly because they can overcome vector neutralization by antibodies against AAV8 formed following AAV8-HBV hepatic delivery. Additionally, AAV2 vectors have been extensively used in human clinical trials and have shown an excellent safety profile. Although conventional single-stranded (ss) AAV2 vector transduces mouse hepatocytes poorly, scAAV2 vectors overcome the rate-limiting second-strand DNA conversion and are able to transduce hepatocytes very efficiently^25,26^. In addition to the vectors carrying individual shRNA cassettes, vectors containing shRNA-1 and -3 combination cassettes were also produced (Fig. 1b,c). A shRNA vector against TGF-β was also generated (Fig. 1d).

**Figure 2:**
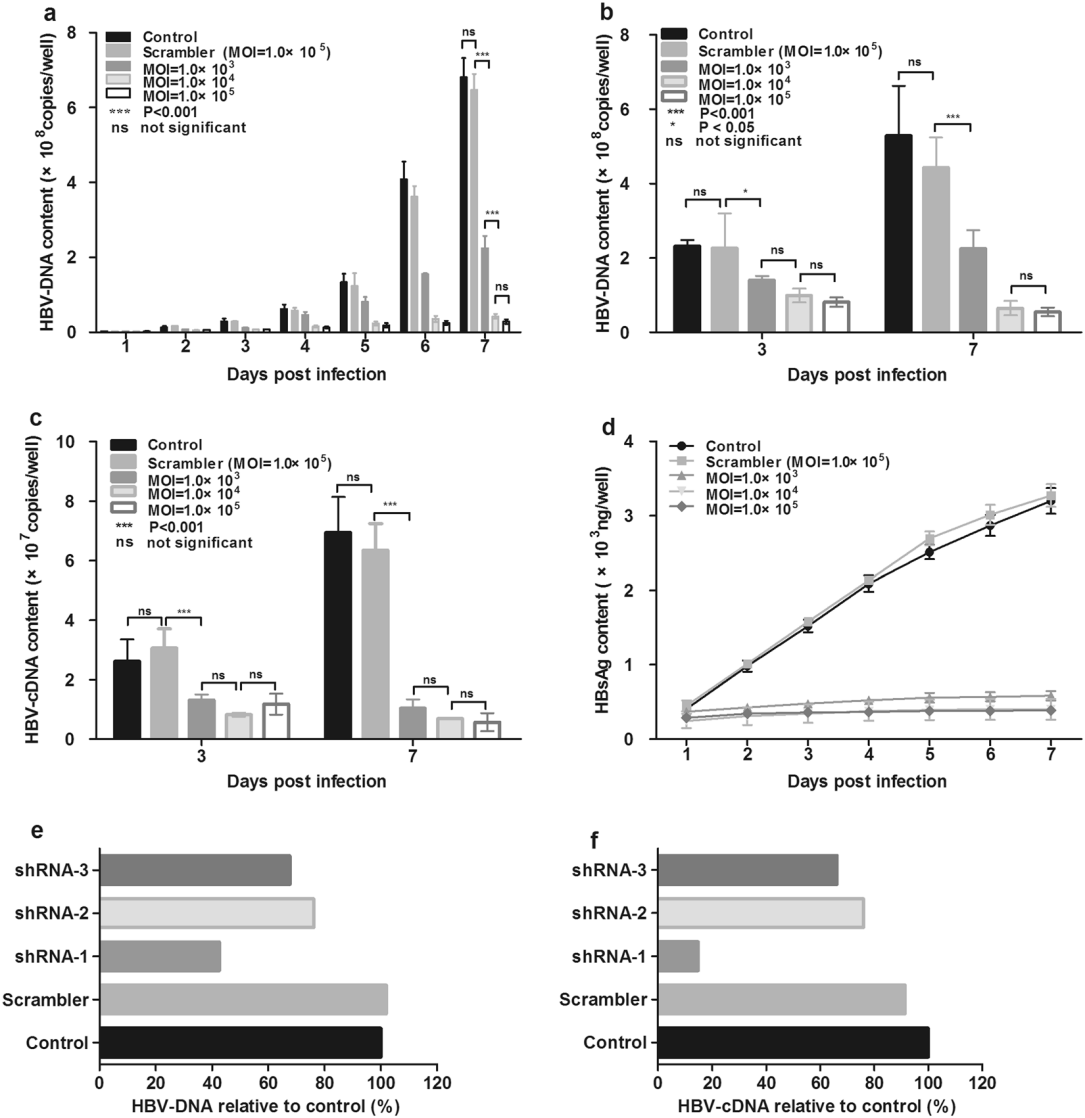
AAV-shRNA-1 inhibits HBV replication, transcription, and HBsAg expression in HepG2.2.15 cells. HepG2.2.15 cells were infected with AAV2-shRNA-1 at a multiplicity of infection (MOI) of 1000, 10000, and 100000. Supernatants and cells were collected separately at the indicated time points post infection. Changes of HBV DNA in supernatants (**a**) and cells (**b**), HBV RNA in cells (**c**), and HBsAg in supernatants (**d**) were monitored during a 7 day period. The inhibitory effects of different AAV-shRNAs at an MOI of 1000 on HBV DNA (**e**) or HBV RNA (**f**) were estimated by the amount of HBV DNA or HBV RNA after each treatment relative to that of the control (%). Control, PBS treatment; scrambler, vector containing the shRNA sequence not targeted to the HBV genome. P < 0.001: significant difference. Ns: the difference was not significant. Data represent the mean ± SD of three independent experiments.

**Figure 3:**
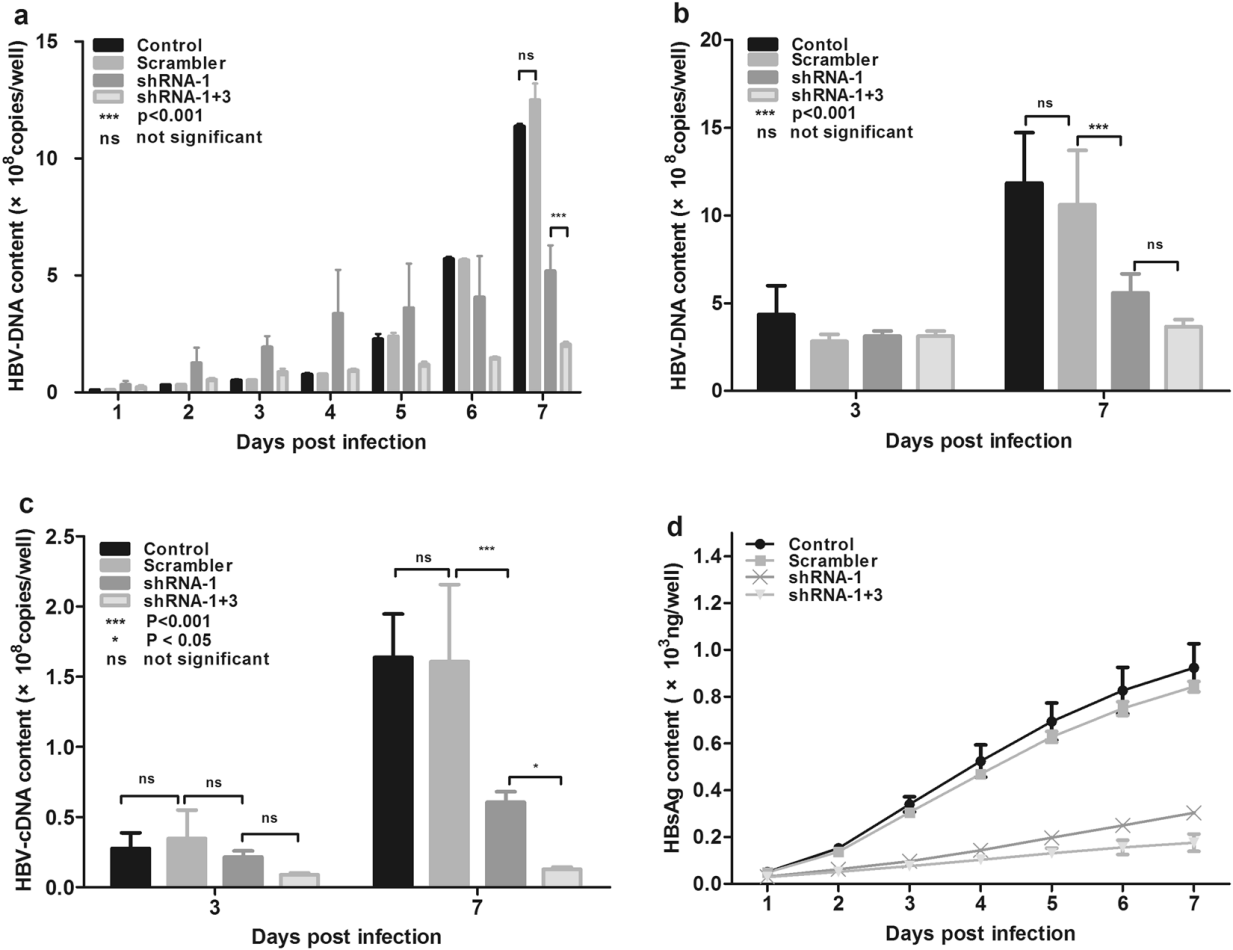
Dual-shRNA has a stronger inhibitory effect on HBV replication than single shRNA in HepG2.2.15 cells. HepG2.2.15 cells were infected with AAV vectors containing single or dual shRNAs at a MOI of 2000. Supernatants and cells were collected separately at the indicated time points post infection. Changes of HBV DNA in supernatants (**a**) and cells (**b**), HBV RNA in cells (**c**), and HBsAg in supernatants (**d**) were monitored during a 7 day period. Control, PBS treatment; scrambler, vector containing the shRNA sequence not targeted to the HBV genome. P < 0.05: significant difference. Ns: the difference was not significant. Data represent the mean ± SD of three independent experiments.

The antiviral effect of the individual shRNAs was first evaluated using HepG2.2.15 cells harboring HBV genome. AAV2-shRNA-1 targeting the S region showed robust inhibition of HBV in a dose-dependent manner, as revealed by the >90% decrease of HBV genomic DNA both in the supernatant and cell lysates at 7 days post infection, when the highest multiplicity of infection (MOI) was used (Fig. 2a,b). This inhibitory effect was also reflected by the 90% decrease of HBV RNA in cells and 90% decrease of HBsAg in the supernatant (Fig. 2c,d). The other two shRNAs targeting a downstream S region, and X antigen, respectively, appeared to be less potent than shRNA-1 for HBV inhibition (Fig. 2e,f) (see Supplementary Figures S1,S2). We then tested whether the combination of two shRNA targeting different HBV coding regions could have a stronger inhibitory effect on HBV than shRNA-1, the most potent individual shRNA. With an MOI of 2.0 × 10^3^ vector genomes (vg)/cell, the vector carrying the combination of shRNA-1 and -3 exhibited significantly higher anti-HBV activity than the shRNA-1 vector, causing 90% reduction of HBV DNA in the medium and approximately 60% reduction in cell lysates at 7 days post infection (Fig. 3a,b). At this time point, the shRNA-1 and -3 combination resulted in an approximately 10-fold reduction of HBV RNA in cell lysates and HBsAg content in the supernatant, which was remarkably better than shRNA-1 treatment alone (Fig. 3c,d). As inhibition of HBV DNA replication is a consequence of RNAi inhibition of HBV RNA which takes time, reduction of HBV DNA in the cell lysates at day 3 post treatment was not obvious. Reduction of HBV DNA at day 7 mainly reflected the inhibition of newly synthesized HBV genome, as the pre-existing HBV genome in the cell line was not the major target of RNAi and was not significantly affected.

### Anti-HBV effect in HBV-persistent mice

**Figure 4:**
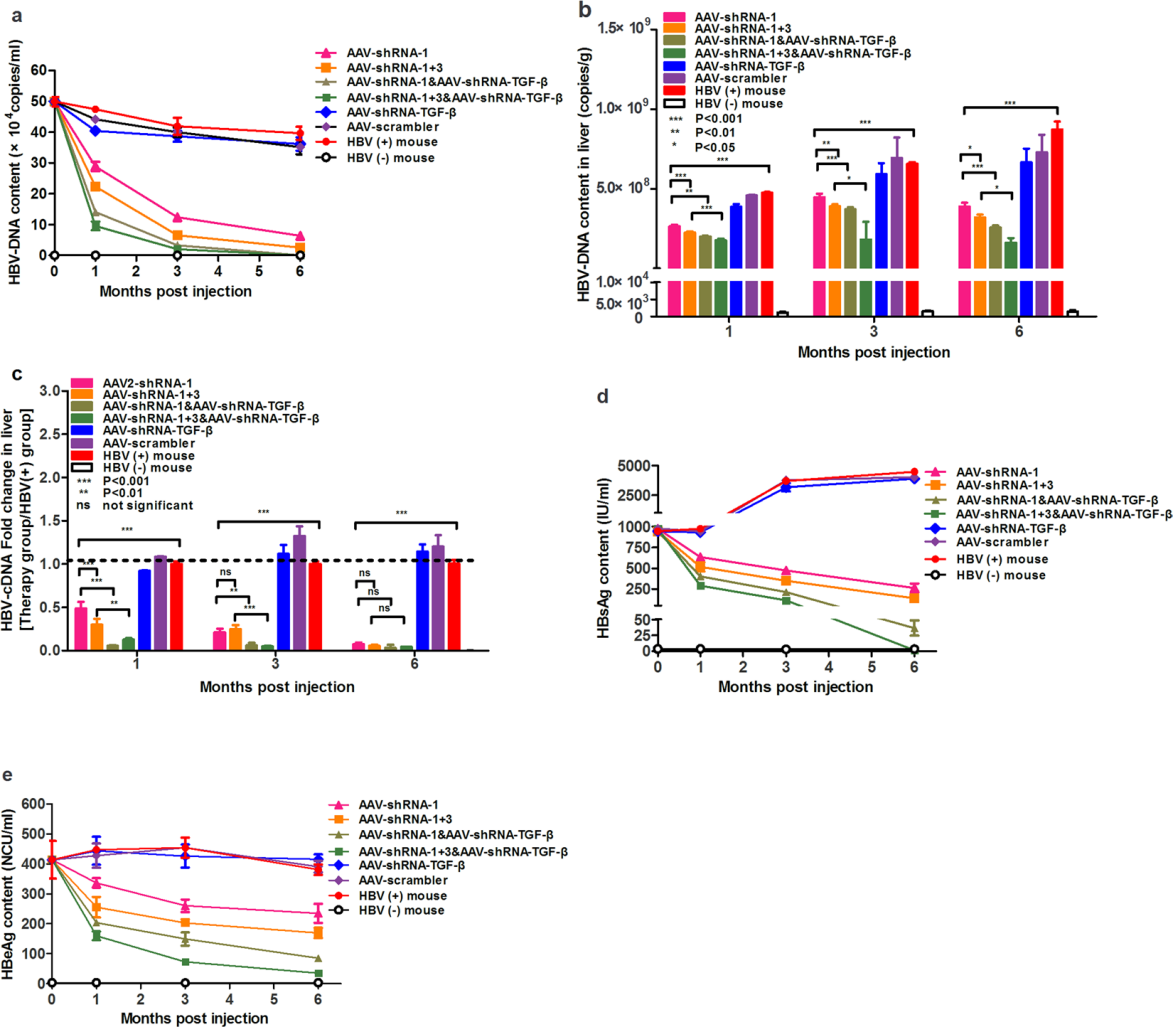
AAV-shRNA inhibits HBV replication and expression in HBV-persistent mice. Mice received AAV2-shRNA treatment, and serum and liver samples were collected at the indicated time points during a 6 month period. HBV DNA levels in serum (**a**) and liver tissues (**b**) were measured by qPCR, and HBV RNA levels were quantified by RT-qPCR (**c**). Serum HBsAg (**d**) and HBeAg (**e**) were measured by ELISA. P < 0.05: significant difference, Data represent the mean ± SD. HBV(+) mice, HBV-persistent mice with administration of PBS; HBV(−) mice, wild-type mice injected with PBS; AAV-scrambler, HBV-persistent mice treated with AAV2-scrambler, a vector carrying a scrambled shRNA sequence not targeted to the HBV genome; AAV-shRNA-1, HBV-persistent mice treated with an AAV2 vector carrying the single shRNA-1 against HBV; AAV-shRNA-1 + 3, HBV-persistent mice treated with an AAV2 vector carrying shRNA-1 and shRNA-3 against HBV; AAV-shRNA-TGF-β, HBV-persistent mice treated with AAV2-shRNA against TGF-β.

The HBV-persistent mouse line established by AAV8-mediated hepatic delivery of the HBV genome was used to evaluate the anti-HBV effect of the shRNAs at 1 month after AAV8-HBV injection. Mice received tail-vein injections of the AAV2-shRNA vectors at a dose of 2 × 10^11^ vg per mouse and were examined for changes of HBV DNA, HBV RNA, HBsAg, and HBeAg in the serum and/or liver tissues during a 6- month period. As TGF-β is an important fibrogenic cytokine that can activate collagen-producing cells^22^, we also tested whether co-administration of shRNA against TGF-β (shRNA-TGF-β) with shRNAs against HBV could act synergistically to inhibit HBV replication. Analysis of HBV replication by quantitative PCR (qPCR) in sera (Fig. 4a) and whole liver lysates (Fig. 4b) showed that AAV-shRNAs against HBV caused a dramatic reduction of the HBV genome content. The combination of shRNA-1 and -3 caused a greater reduction of HBV DNA than shRNA-1 alone during the entire 6 month period, consistent with our *in vitro* study (Fig. 3). Although the shRNA against TGF-β did not inhibit HBV when used alone, it markedly enhanced the anti-HBV effect of shRNA-1 and shRNA-1 + 3, especially at early time points following vector administration. Similar HBV inhibition by these shRNAs was observed in liver lysates, although to a lesser extent than in the sera. Assessment of HBV RNA changes in liver lysates confirmed that shRNA-TGF-β enhanced the anti-HBV effect of shRNA-1 and shRNA-1 + 3 (Fig. 4c). The serum levels of HBsAg and HBeAg declined rapidly in shRNA-1 and shRNA-1 + 3 treated mice and continued to decline during the 6 month period. In mice treated with AAV-shRNA-1 + 3 and AAV-shRNA-TGF-β, HBsAg decreased to undetectable levels at 6 months after administration (Fig. 4d). A similar trend was observed in the reduction of serum HBeAg, although to a lesser extent than that of HBsAg in the same mice (Fig. 4e).

**Figure 5:**
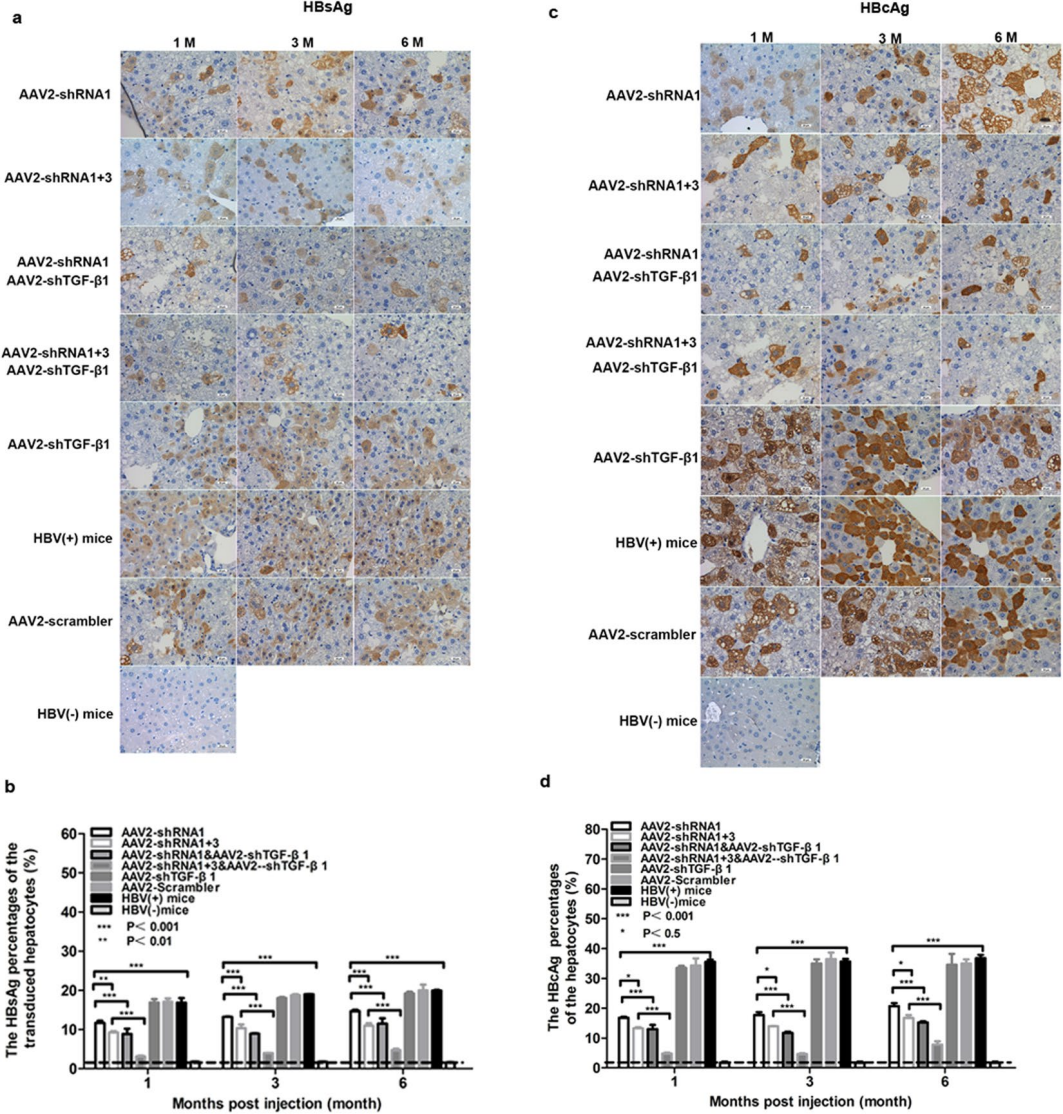
AAV-shRNA treatment decreases HBsAg and HBcAg in hepatocytes of HBV-persistent mice. Serum and liver samples of mice treated with AAV2-shRNA were collected at the indicated time points during a 6 month period. Liver sections were fixed and stained for HBsAg (**a**) and HBcAg (**c**). Ten random fields were selected per slide, and the percentages of HBsAg- (**b**) and HBcAg-positive (**d**) hepatocytes were quantified using Image-Pro Plus. P < 0.05: significant difference, Data represent the mean ± SD.

To directly examine the clearance of HBV from hepatocytes, immunohistochemical staining for the HBV antigen and quantification of the results were performed in mouse livers. Consistent with serum level changes, HBsAg decreased markedly in hepatocytes in mice treated with shRNA-1 or shRNA-1 + 3, and shRNA-TGF-β significantly enhanced this effect. Specifically, treatment with shRNA-TGF-β together with shRNA(s) against HBV reduced HBsAg to a level similar to that in mice with no HBV replication (Fig. 5a,b). A similar reduction trend was observed for HBcAg in hepatocytes (Fig. 5c,d), although to a lesser extent.

**Figure 6:**
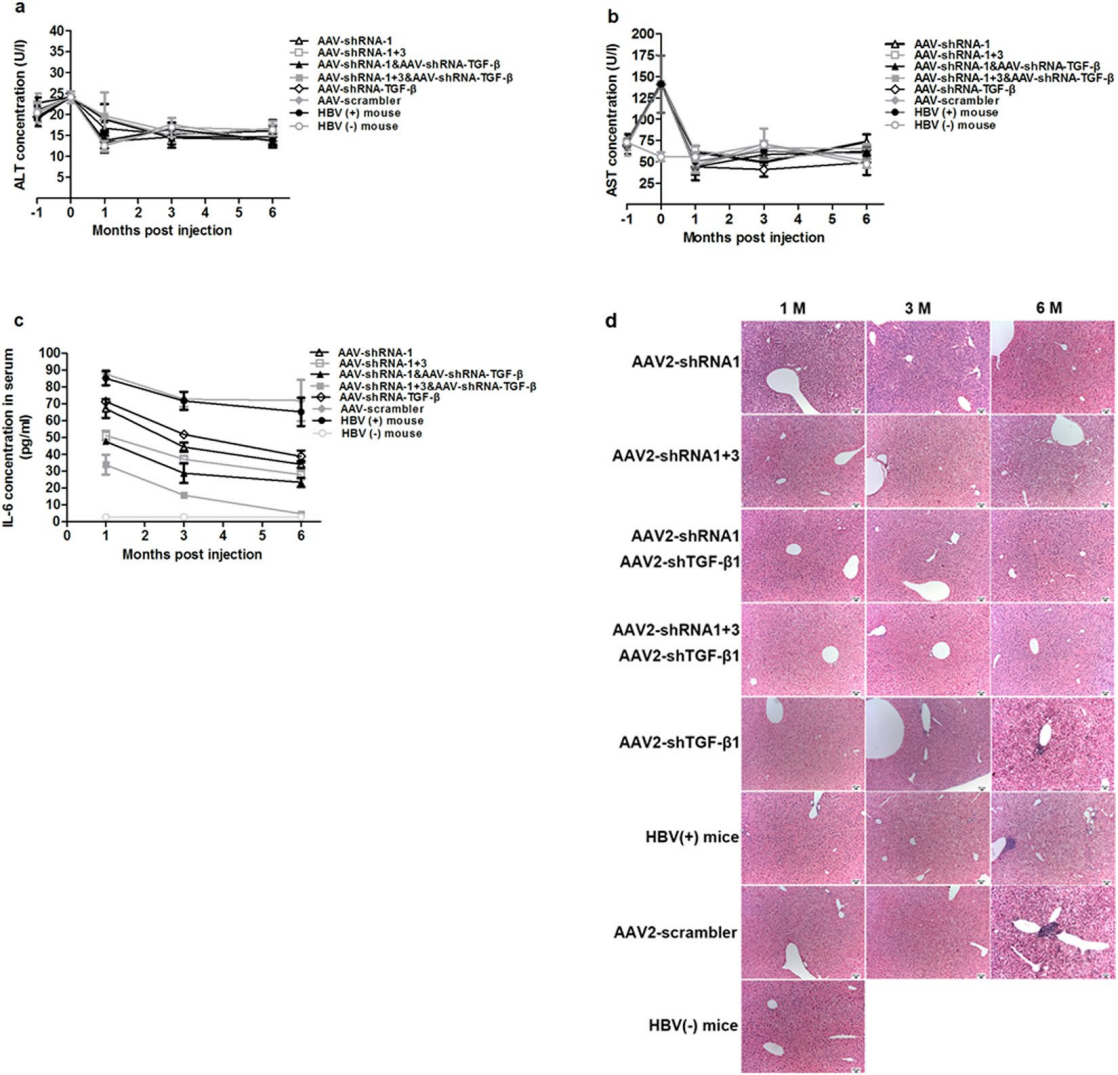
AAV-shRNA treatment improves liver tissue histology without inducing acute inflammation. Mice received AAV2-shRNA treatment, and serum and liver samples were collected at the indicated time points during a 6 month period. (**a–c**) Time-course changes of serum levels of ALT (**a**), AST (**b**), and IL-6 (**c**). (**d**) Liver sections stained with hematoxylin and eosin. Data represent the mean ± SD.

Overexpression of certain shRNAs can induce acute cytotoxicity^27^. To test the toxicity of the AAV-shRNA vectors used in the present study, the sera of treated mice were examined for transaminase concentrations. As indicated in Fig. 6a and b, administration of AAV-shRNAs did not increase serum ALT and AST levels during the 6 month period, indicating that no obvious acute inflammation was induced. Other inflammatory factors, such as IFN-γ, TNF-α, IL-10, and IL-6, were also tested. IL-6 levels were significantly higher in all groups of HBV-persistent mice than in HBV-negative mice at the 1 month time point. Although the IL-6 level continued to decline in almost all treatment groups during the 6 month period, mice receiving co-administration of AAV2-shRNA-TGF-β and AAV2-shRNA-1 + 3 showed the lowest IL-6 levels at all time points, with almost undetectable levels at 6 months, similar to those in the HBV-negative mice (Fig. 6c). The levels of other cytokines did not differ significantly between HBV-persistent mice and HBV-negative mice (data not shown). Animals treated with AAV-shRNAs did not show other symptoms of systemic toxicity (data not shown). To test chronic liver injury, histopathological changes of liver tissues were examined by hematoxylin and eosin (H&E) staining (Fig. 6d). Although the vast majority of hepatocytes appeared histologically normal during the first 3 months after injection, hepatic necrosis was observed at 6 months in the AAV8-HBV, AAV-scrambler, and AAV-shRNA-TGF-β injected mice, with destruction of hepatic lobular structure and macrovesicular steatosis degeneration. Morphological improvements were observed in AAV-shRNA-1 and AAV-shRNA-1 + 3 treated mice, whether AAV2-shRNA-TGF-β was co-administered or not.

### Antifibrotic effects of AAV-shRNAs targeting HBV and TGF-β

**Figure 7:**
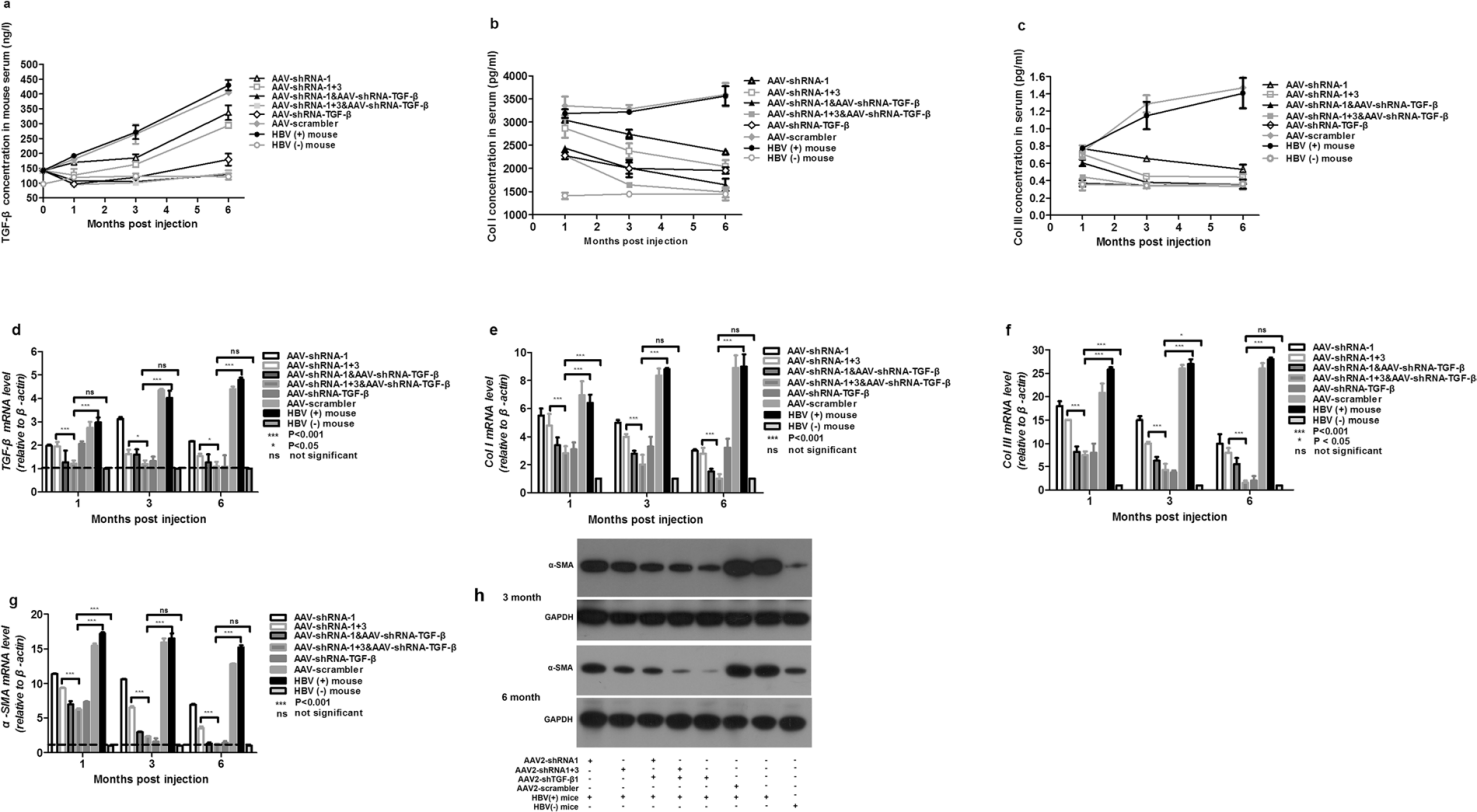
AAV-shRNA treatment decreases the expression of liver fibrosis biomarkers in HBV-persistent mice. Mice received AAV2-shRNA treatment, and serum levels of TGF-β (**a**), collagen I (**b**), and collagen III (**c**) were measured by ELISA during a 6 month period. (**d**–**g**) Reverse transcription quantitative PCR measurements of Tgf-β1 (**d**), Col I (**e**), Col III (**f**), and α-SMA (**g**) mRNA levels in the liver. (**h**) Western blot analysis of α-SMA in liver samples. Statistical analyses were performed using a two-way analysis of variance. P < 0.05: significant difference. Ns: the difference was not significant. Data represent the mean ± SD (n = 4).

Our previous study indicated that AAV-HBV injection can induce HBV chronic replication and liver fibrosis^20^. To test the antifibrotic effects of the AAV-shRNA vectors, a number of fibrogenic biomarkers and extracellular matrix (ECM) proteins were examined after administration of AAV-shRNAs. As one of the most potent fibrogenic cytokines, TGF-β plays an important role in the initiation and maintenance of hepatic fibrogenesis. Consistent with our previous observation^20^, the level of serum TGF-β was continuously elevated in the HBV-persistent mice and was not altered by AAV-scrambler vector administration (Fig. 7a). Injection of AAV-shRNA-TGF-β remarkably reduced the level of serum TGF-β at all time points during the 6 month period. Administration of AAV-shRNA-1 or AAV-shRNA-1 + 3 also decreased the rate of TGF-β elevation, albeit less efficiently than AAV-shRNA-TGF-β. Co-administration of AAV-shRNA-TGF-β with shRNA-1 or shRNA-1 + 3 had the strongest effect, reducing TGF-β to nearly normal levels. A similar pattern of TGF-β mRNA changes was observed in liver tissues after vector treatment (Fig. 7d). To quantify the major ECM proteins, serum collagen I (Col I) and III (Col III) levels were determined in these mice. As shown in Fig. 7b and c, serum Col I and Col III were either maintained at or gradually increased to a high level in untreated and AAV-scrambler treated HBV-persistent mice during the 6 month period. By contrast, these levels were significantly reduced in AAV-shRNA-1, AAV-shRNA-1 + 3, and AAV-shRNA-TGF-β treated mice. Co-administration of AAV-shRNA-TGF-β with shRNA-1 or shRNA-1 + 3 vector caused the greatest reduction of both serum Col I and Col III. Similar patterns of reduction of the collagen I (Fig. 7e) and III (Fig. 7f) mRNAs were observed in the livers of mice receiving different AAV-shRNA treatments. Upregulated α-SMA is a biomarker of activated HSCs in the fibrogenic liver. The combination of AAV-shRNA-TGF-β with anti-HBV shRNA vectors significantly reduced α-SMA expression at both the transcription (Fig. 7g) and translation levels (Fig. 7h).

**Figure 8:**
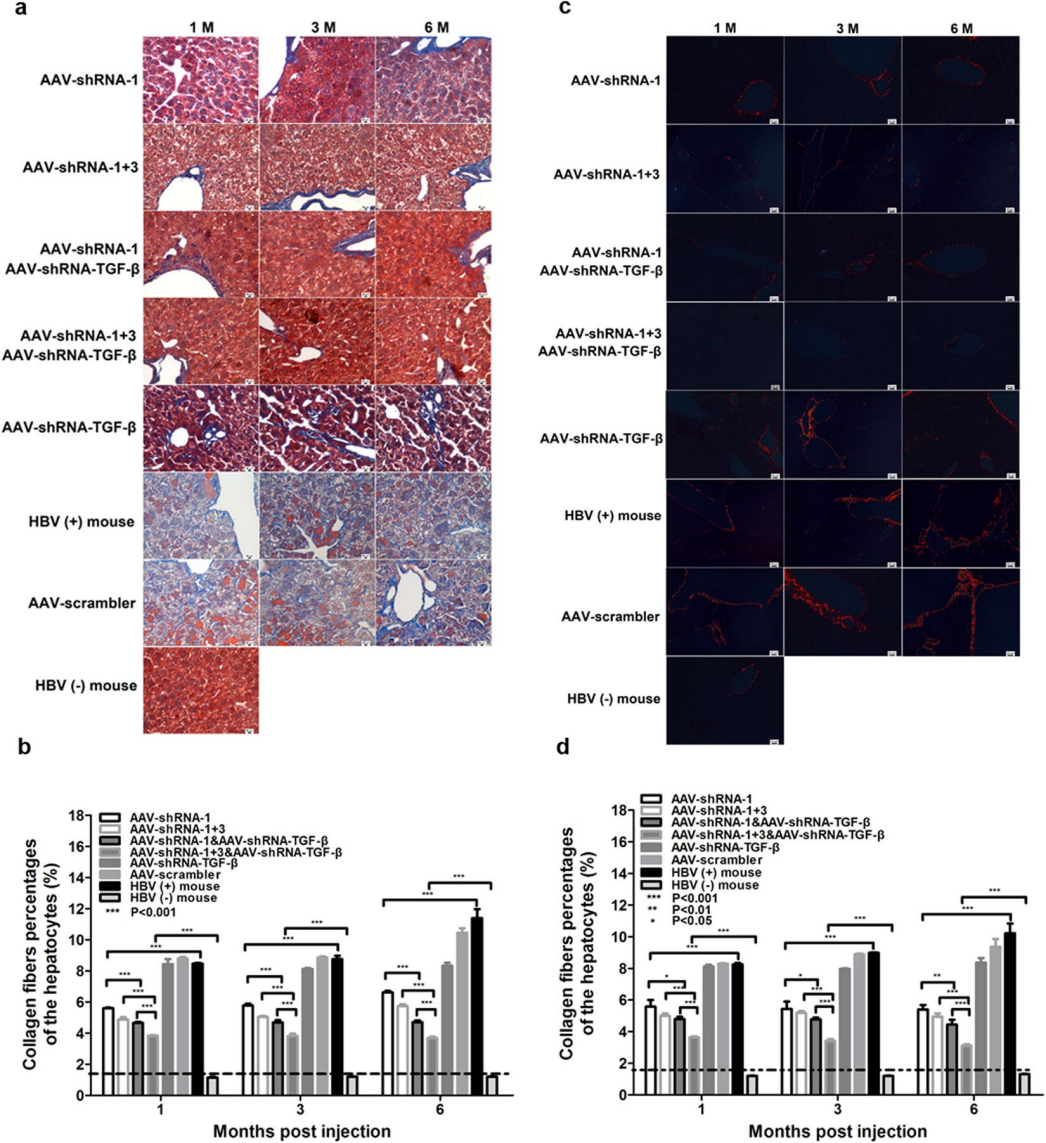
AAV-shRNA treatment attenuates collagen deposition and liver fibrosis in HBV-persistent mice. Mice received AAV2-shRNA treatment and were euthanized at 6 months post vector administration. Collagen deposition and collagen fibers were examined by Masson’s trichrome (**a**) and Sirius red staining (**c**) of the liver sections, respectively. Sirius red staining was observed under polarizing microscope. Ten random fields were selected per slide, and the percentages of hepatocytes with collagen deposition (**b**) and collagen fibers (**d**) were quantified using Image-Pro Plus. P < 0.05: significant difference, Data represent the mean ± SD.

To evaluate the morphological changes of liver tissues after shRNA treatment, Masson’s trichrome staining was performed to detect collagen deposition (Fig. 8a,b). Extensive collagen deposition was observed in liver tissues of untreated and AAV-scrambler treated HBV-persistent mice during the 6 month period. Although administration of AAV-shRNA-TGF-β alone only slightly inhibited collagen accumulation, mice receiving other shRNA treatments showed a significant reduction in collagen accumulation. Particularly, co-administration of AAV-shRNA-TGF-β with other shRNA vectors resulted in the lowest collagen accumulation. Sirius red staining was also performed to indicate changes of fiber production in treated livers (Fig. 8c,d). Fibrous expansion of portal areas with marked bridging was evident in untreated, AAV-scrambler treated, and AAV-shRNA-TGF-β treated mice. By contrast, the livers of AAV-shRNA-1 and AAV-shRNA-1 + 3 treated mice exhibited a much lower level of fibers and little fibrous expansion of portal areas with short fibrous septa. Co-administration of AAV-shRNA-TGF-β with AAV-shRNA-1 or AAV-shRNA-1 + 3 resulted in an even stronger inhibition of fiber production. Taken together, these results indicated severe collagen deposition and fibrogenesis in the livers of HBV-persistent mice, which were dramatically attenuated by the administration of AAV-shRNAs, especially by the injection of shRNA vectors targeting both HBV and TGF-β.

## Discussion

HBV infection remains a serious health care problem, with up to 400 million carriers and approximately one million deaths annually resulting from cirrhosis, liver failure, or HCC^28^. Current therapies including IFNs and nucleoside/nucleotide analogs are not effective at clearing HBV completely from the livers of affected patients. There is an urgent need to develop novel treatment regimens with better responses, shorter duration, and lower resistance and reactivation rates.

RNAi is a conserved sequence-dependent gene silencing process in which RNA molecules inhibit gene expression by transcriptional or post-transcriptional gene silencing. In a number of studies, synthetic or expressed RNA sequences carried by viral or non-viral vectors have shown promising results in inhibiting HBV replication^29–31^. In particular, a clinical study showed that ARC 520, which consists of two synthesized cholesterol-conjugated siRNAs and a hepatocyte-targeting peptide, was safe, well-tolerated, and able to reduce HBsAg significantly in patients. To avoid repeated administration, in the present study, we evaluated three shRNAs carried by the AAV2 vector that were persistently expressed. Our *in vitro* results (Fig. 2) indicated that AAV-shRNA-1 had the greatest antiviral activity, with at least 90% inhibition efficiency at the replication, transcription, or translation level. Since single siRNA administration could be ineffective to inhibit pre-existing mutant HBV genomes^32^, the combination of multiple shRNAs targeting different HBV codon regions was suggested to overcome this problem and has yielded superior antiviral effects^33^. In the present study, the antiviral efficacy of AAV-shRNA-1 + 3 was 2-fold higher than that of a single AAV-shRNA at the DNA and protein levels, whereas a 5-fold higher efficacy was obtained at the RNA level with the use of a dual-shRNA vector. The antiviral effects of these shRNAs were also assessed in the HBV-persistent mouse model recently established by our group^20^. As indicated in Fig. 4, HBV DNA and antigen were significantly reduced by administration of AAV-shRNAs against HBV. The viral load in the serum was reduced by approximately 50-fold, and HBsAg and HBeAg were reduced to almost undetectable levels by the administration of AAV-shRNA-1 + 3. We noticed that the reduction of HBV DNA in the liver was not as remarkable as that in the serum. This could be related to the nature of the HBV mouse model we used, in which HBV-containing AAV genome can exist in the nuclear of hepatocyte as an episomal form persistently. ShRNAs against HBV can inhibit HBV replication by suppressing HBV RNA but do not affect the AAV-delivered HBV genome. As the HBV DNA in mouse liver contains both the newly formed HBV DNA and the existing AAV-HBV genomes while the serum HBV DNA is completely newly synthesized, the almost undetectable level of serum HBV DNA after shRNA treatment (Fig. 4a) indicated the potency of our approach. It is also interesting that the reduction of HBV antigen levels was different between the serum and the liver, which could be due to the different half-life in the serum and the liver. HBV antigens can be cleared rapidly in the blood circulation, but longer time could be needed for its clearance in hepatocytes. Collectively, these results demonstrated that the dual-AAV-shRNAs have potent antiviral effects both *in vitro* and *in vivo*.

TGF-β plays critical roles not only in the progression of persistent HBV infection but also in the induction of liver fibrosis, cirrhosis, and HCC following HBV infection^34–36^. In our HBV-persistent mouse model, serum TGF-β expression increased by approximately 4-fold at 6 months after injection (Fig. 7a). Previous studies reported contradictory results regarding the effects of TGF-β on regulating HBV infection. Antiviral effects of TGF-β mediated by the upregulation of cellular HNF-4^37^ or microRNA expression^38^ were reported. However, it was also reported that elevated TGF-β could inhibit the immune response rather than increase pro-inflammatory cytokines that favors HBV-persistent infection^21^. The major sources of TGF-β in the mouse liver are thought to be the damaged hepatocytes and Kupffer cells. Although report of AAV transduction to mouse Kupffer cells is lacking, it was shown that a significant amount AAV can be taken by rat Kupffer cells via scavenger receptor A (SR-A) following vector liver delivery^39^. Therefore, it is likely that mouse Kupffer cells can be efficiently transduced by scAAV2 as well. In the present study, shRNA-mediated knockdown of TGF-β expression had no obvious effect on HBV replication. By contrast, co-injection of AAV-shRNA-TGF-β with AAV-shRNAs against HBV enhanced the antiviral activity (Fig. 4). HBsAb levels were analyzed by ELISA in mice receiving AAV-shRNA treatment (see Supplementary Figure S3). HBsAg seroconversion was achieved in only five mice, and all five mice were treated with AAV-shRNA-TGF-β. HBsAb reached high levels in two mice in the AAV-shRNA-1 + 3 and AAV-shRNA-TGF-β groups. This suggests that an unknown pathway of TGF-β was involved in the inhibition of HBV production. Previous studies reported that high levels of circulating HBV viral antigen could induce immune tolerance, leading to the inhibition of anti-HBsAb production and virus-specific CD8 + T cell responses^40,41^. In addition to enhancing the antiviral efficacy of AAV-shRNA-1 + 3, AAV-shRNA-TGF-β administration may help immune restoration. Additional study is needed to clarify the role of TGF-β in this seroconversion.

Liver fibrosis is caused by excessive accumulation of ECM proteins, including collagen produced by collagen-producing cells when activated by fibrogenic cytokines^42,43^. TGF-β is a key cytokine involved in liver fibrosis that stimulates hepatic stellate cells to transdifferentiate into myofibroblast-like cells^44^, resulting in the production of ECM proteins. TGF-β also inhibits the degradation of ECM proteins. Preventing the deposition of collagen is one of the strategies of antifibrotic therapies. A previous study demonstrated that blocking the synthesis and/or signaling pathways of TGF-β can decrease liver fibrosis in animal models^45^. Similarly, in the present study, AAV-shRNA-TGF-β administration significantly downregulated fibrosis markers in the serum and/or liver, including TGF-β, Collagen I, Collagen III, and α-SMA. However, no morphological improvement was achieved (Fig. 8c). On the other hand, administration of AAV-shRNA-1 + 3 not only exhibited robust anti-HBV activity but also demonstrated efficient antifibrotic activity, as revealed by both the reduction of serum fibrosis markers (Fig. 7) and improved tissue structure (Fig. 8). These results indicate that removal of the causative agent is an effective strategy for treating liver fibrosis, while inhibition of the TGF-β pathway alone is not. However, the combination of these two strategies resulted in better antifibrotic effects, as revealed by a further reduction of serum fibrosis markers and the remarkable change in the hepatic parenchyma from severe fibrosis to moderate fibrosis. Taken together, these results demonstrated the advantages of the combinatorial use of shRNAs against both HBV and TGF-β in preventing liver fibrosis.

In summary, the present study showed for the first time the persistent anti-HBV and antifibrosis effects of RNAi in a HBV-persistent mouse model mimicking human chronic HBV infection. The AAV-delivered shRNAs against HBV and TGF-β could be developed into a viable treatment for human HBV infection. Future studies should include screening shRNAs with high specificity, better silencing efficiency, and fewer off-target effects. These improvements could be achieved by adopting a few mismatches of the shRNA with the unintended mRNA, using embedded shRNA in an artificial microRNA, co-expressing Argonaute-2, or co-delivering a decoy directed against the shRNA sense strand^46^. However, all these methods do not affect viral cccDNA which was the key element for cure of chronic HBV infection. Utilizing gene editing methods, such as clustered regularly interspaced short palindromic repeats (CRISPR) with CRISPR-associated (Cas) nucleases, engineered zinc finger nucleases (ZFNs) and transcription activator-like effector nucleases (TALENs), to mutate cccDNA supplies the new direction for permanently inactivation of HBV gene expression^47^.

## Materials and Methods

### Design of siRNAs

HBV target sequences were selected in regions overlapping the viral 3.5, 2.4, and 2.1 kb RNAs, according to the parameters indicated on the siRNA Target Finder website (www.ambion.com).

### Construction of recombinant pAAV-shRNAs

Recombinant pshRNA plasmids were constructed using standard molecular cloning techniques. Four pairs of oligonucleotide duplexes were synthesized by Thermo Fisher Scientific (see Supplementary Table S1). A general strategy for constructing pSC-H1-shRNA expression vectors involved ligating an annealed oligonucleotide duplex into the *Bg*lII/*Hind*III restriction sites of the pSC-H1 vector (containing human RNA polymerase-III H1 RNA gene promoter). For dual-shRNA vector construction, the fragment H1-shRNA-3 (digested from pSC-H1-shRNA-3 by *EcoR*I/*Hind*III) was inserted into the *EcoR*I site of pSC-H1-shRNA-1, resulting in pSC-H1-shRNA-1 + 3. These shRNA expression cassettes were inserted between the two inverted terminal repeats (ITRs) of the pSSV9 plasmids, resulting in pAAV-shRNAs.

### Cell lines

The 293T and Huh7 cell lines were maintained in complete medium (Dulbecco’s Modified Eagle’s Medium supplemented with heat-inactivated 10% fetal calf serum and 1% penicillin-streptomycin solution) at 37 °C in a humidified incubator with 5% CO_2_. The HepG2.2.15 cell line (with the HBV *ayw* genome) was maintained as described above, but the medium was additionally supplemented with the antibiotic G418 (200 μg/ml).

### AAV vector production and purification

AAV vectors carrying shRNAs targeting HBV and TGF-β were packaged with AAV2, and AAV vectors carrying HBV genome was packaged with AAV8 which was efficient for liver transduction^48^, and vectors were produced in 293 T cells using a triple-plasmid transfection protocol and purified by ultra-centrifugation on CsCl gradients^49^. Vector titers were determined by qPCR.

### *In vitro* study

HepG2.2.15 cells were trypsinized and seeded in six-well culture plates at 5 × 10^5^ cells per well. Twenty-four hours later, the cells were infected with AAV-shRNA vectors at the indicated MOI. After incubation at 37 °C for 3 h, the supernatants were replaced with fresh complete medium. The supernatants of AAV-shRNA-treated HepG2.2.15 cells were harvested every 24 h, and the cells were collected at 3 and 7 days post infection.

### Animal study

Normal C57BL/6 mice (aged 6–8 weeks; Vitalriver, Beijing, China) were bred and maintained at the Laboratory Animal Facility of the Institute of Laboratory Animal Sciences, Chinese Academy of Medical Science, Beijing. Animal care and procedures were performed in accordance with the Guide for the Care and Use of Laboratory Animals, which was approved by the Institutional Animal Care and Use Committee at the Chinese Academy of Medical Science. Mice were injected with the AAV8-HBV1.2 vector [2 × 10^11^ vector genome equivalents (vg)] in 200 µl of phosphate-buffered saline (PBS) via the tail vein. One month post injection, the serum for the ELISAs and qPCR was prepared by tail bleeding and collected in heparinized capillary tubes using standard methods. The HBsAg-, HBeAg-, and HBV DNA-positive mice were designated as HBV-positive mice and injected with AAV-shRNAs, AAV-shRNA-TGF-β, and AAV-scrambler (2 × 10^11^ vg), respectively. The serum for the ELISAs was prepared from tail bleeding as described above. After dilution with PBS, the serum HBsAg and HBeAg concentrations were measured using an ARCHITECT HBsAg (or HBeAg) Reagent Kit (Abbott GmbH&Co.KB, Wiesbaden, Germany). Serum alanine aminotransferase (ALT) and aspartate aminotransferase (AST) levels were analyzed using commercially available colorimetric assays (R&D Systems, Minneapolis, MN). To collect the livers for the immunohistochemical and nucleic acid analyses, mice were anesthetized using 2.5% avertin and perfused transcardially with cold PBS (pH 7.4), followed by 4% paraformaldehyde in phosphate buffer (0.1 mol/l, pH 7.4). Intrahepatic HBcAg and HBsAg were visualized by immunohistochemical staining of OCT-embedded tissues using rabbit anti-HBc and anti-HBs antibodies (Dako, Carpinteria, CA), respectively, and the Envision HRP (diaminobenzidine) system (Dako, Carpinteria, CA). Ten random fields were selected per slide, and the percentages of HBsAg- and HBcAg-positive hepatocytes were quantified using Image-Pro Plus software (Media Cybernetics, Rockville, MD). The liver sections were also examined by light microscopy after standard H&E and Masson’s trichrome staining. Sirius red staining of liver sections was observed using a polarizing microscope.

### qPCR and reverse transcription qPCR (RT-qPCR)

Serum or cellular DNA was extracted using DNeasy Blood and Tissue kits (Qiagen, Hilden, Germany) according to the manufacturer’s instructions and stored at −80 °C prior to PCR analyses. A qPCR standard curve was generated using 10-fold dilutions of the SSV9-1.2HBV plasmid (1.0 × 10^3^–1.0 × 10^9^ copies/ml). To measure the mRNA levels of HBV, α*-SMA*, and *Tgf-β1*, *Col I*, *and Col III*, total RNA was isolated using a RNeasy Mini kit (Qiagen, Hilden, Germany) and reverse transcribed using a First Strand cDNA Synthesis kit (Toyobo, OSAKA, Japan). Quantitative PCR was performed in triplicate in 96-well optical reaction plates using an ABI 7900 Sequence Detection System (Applied Biosystems, Foster City, CA) and SYBR Green I PCR mix (Roche Diagnostics, Indianapolis, IN), as previously described^20^. The primer sequences are shown in Supplementary Table S2.

### ELISA

The levels of TGF-β1, collagen I, and collagen III in the liver and serum samples were determined using commercially available ELISA kits including Mouse TGF-β1 Quantikine ELISA kit, Mouse Collagen type I (Col I) ELISA kit, and Mouse Collagen type III (Col III) ELISA kit (R&D Systems, Minneapolis, MN). The serum level of TGF-β1 was also determined using a commercially available ELISA kit (R&D Systems, Minneapolis, MN).

### Statistical analysis

Data were expressed as the mean ± SD. Statistical analysis was performed using two-way analysis of variance (ANOVA, Graphpad prism 5) to determine statistically significant differences between groups. P < 0.05 was considered statistically significant.

## Electronic supplementary material

Supplementary PDF File
