# Author Correction: Enhanced antiviral and antifibrotic effects of short hairpin RNAs targeting HBV and TGF-β in HBV-persistent mice

**DOI:** 10.1038/s41598-018-21512-9

**Published:** 2018-03-06

**Authors:** Lei Ye, Fangming Kan, Tao Yan, Jiaqi Cao, Leiliang Zhang, Zhijian Wu, Wuping Li

**Affiliations:** 1 MOH Key Laboratory of Systems Biology of Pathogens, Institute of Pathogen Biology, Chinese Academy of Medical Sciences & Peking Union Medical College, Beijing, 100730 China; 2 Ocular Gene Therapy Core, National Eye Institute, NIH, Bethesda, Maryland 20892 USA

Correction to: *Scientific Reports* 10.1038/s41598-017-04170-1, published online 20 June 2017

The original PDF version of this Article contained an error in the order of corresponding authors. This has now been corrected in the PDF version of this Article.

