# Supplementary material for: Enhanced antiviral and antifibrotic effects of short hairpin RNAs targeting HBV and TGF-β in HBV-persistent mice
Source: Sci Rep. 2017 Jun 20;7:3860. doi: 10.1038/s41598-017-04170-1 (PMC5478661; doi:10.1038/s41598-017-04170-1)
Supplement: Supplementary file 1 — Supplementary PDF File [file 41598_2017_4170_MOESM1_ESM.pdf]

Supplementary Information

Enhanced antiviral and antifibrotic effects of short hairpin RNAs targeting HBV and TGF-β in HBV-persistent mice

Lei Ye[1, £], Fangming Kan[1, £], Tao Yan[1], Jiaqi Cao[1], Leiliang Zhang[1], Zhijian Wu[2, §], Wuping Li[1,§]

MOH Key Laboratory of Systems Biology of Pathogens, Institute of Pathogen Biology, Chinese Academy of Medical Sciences & Peking Union Medical College, Beijing, 100730, China[1.] Ocular Gene Therapy Core, National Eye Institute, NIH, Bethesda, Maryland 20892, USA[2]

[£]Co-first author

[§]Corresponding author

Tel: 86-10-67837970

Correspondence should be addressed to W.L.(liwuping@ipbcams.ac.cn) and Z.W. (wuzh@mail.nih.gov)

Supplementary Information Inventory

Supplementary Figures S1-S3

Supplementary Tables S1-S2

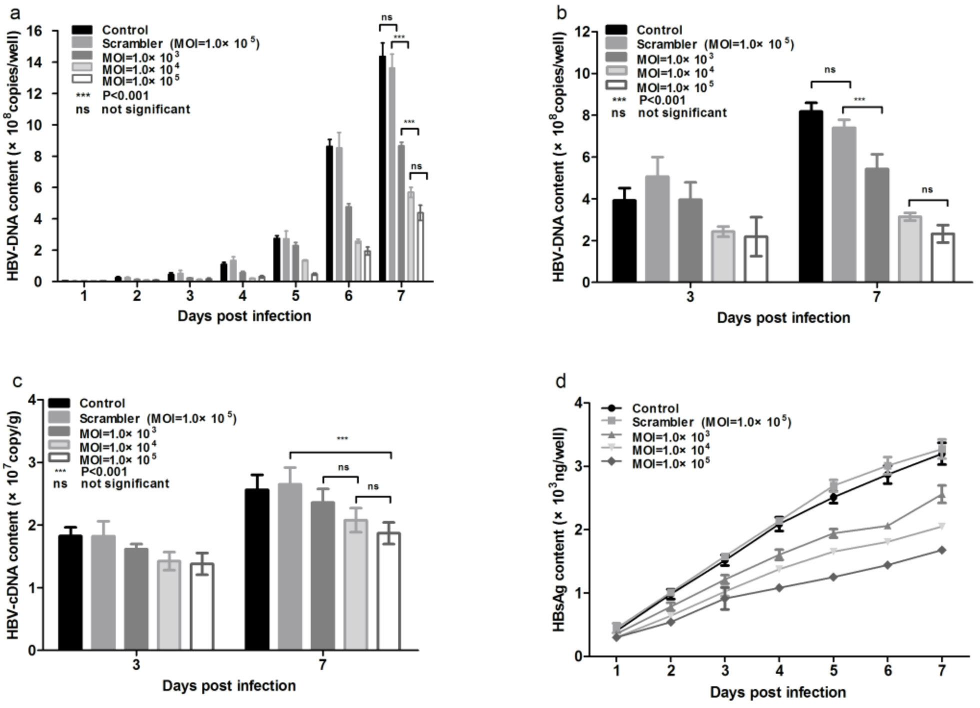

Supplementary Figure S1. AAV-shRNA-2 inhibits HBV replication, transcription, and HBsAg expression in HepG2.2.15 cells.

HepG2.2.15 cells were infected with AAV2-shRNA-2 at a multiplicity of infection (MOI) of 1000, 10000, and 100000. Supernatants and cells were collected separately at the indicated time points post infection. Changes of HBV DNA in supernatants (a) and cells (b), HBV RNA in cells (c), and HBsAg in supernatants (d) were monitored during a 7 day period. Control, PBS treatment; scrambler, vector containing the shRNA sequence not targeted to the HBV genome. P<0.001: significant difference; Ns: the difference was not significant. Data represent the mean ± SD of three independent experiments.

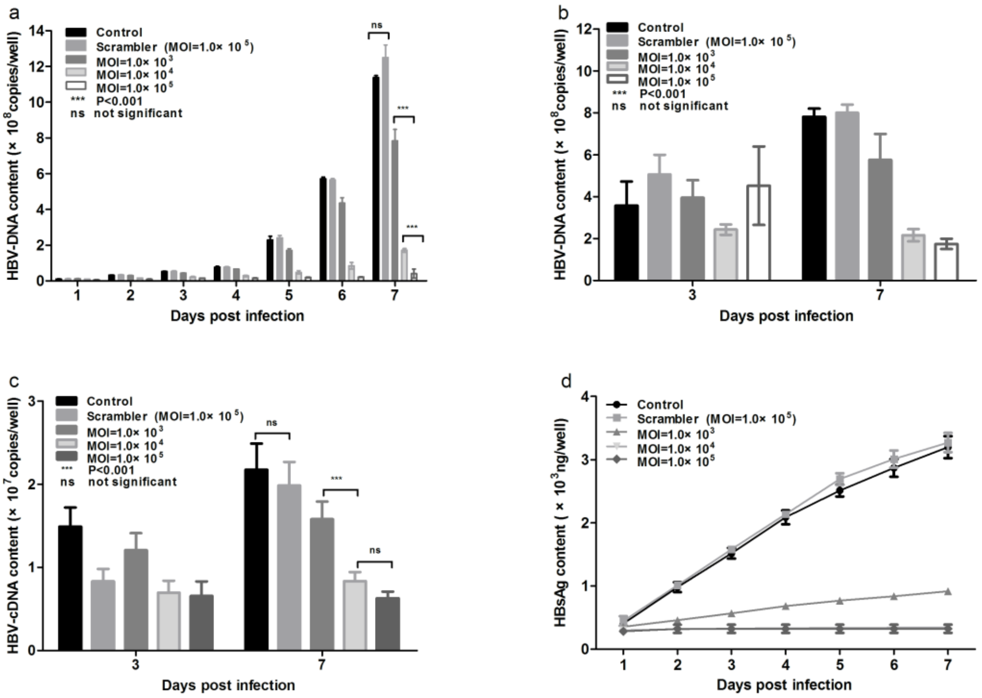

Supplementary Figure S2. AAV-shRNA-3 inhibited HBV replication, transcription, and HBsAg expression in HepG2.2.15 cells.

HepG2.2.15 cells were infected with AAV2-shRNA-3 at a multiplicity of infection (MOI) of 1000, 10000, and 100000. Supernatants and cells were collected separately at the indicated time points post infection. Changes of HBV DNA in supernatants (a) and cells (b), HBV RNA in cells (c), and HBsAg in supernatants (d) were monitored during a 7 day period. Control, PBS treatment; scrambler, vector containing the shRNA sequence not targeted to the HBV genome. $P<0.001$: significant difference; Ns: the difference was not significant. Data represent the mean ± SD of three independent experiments.

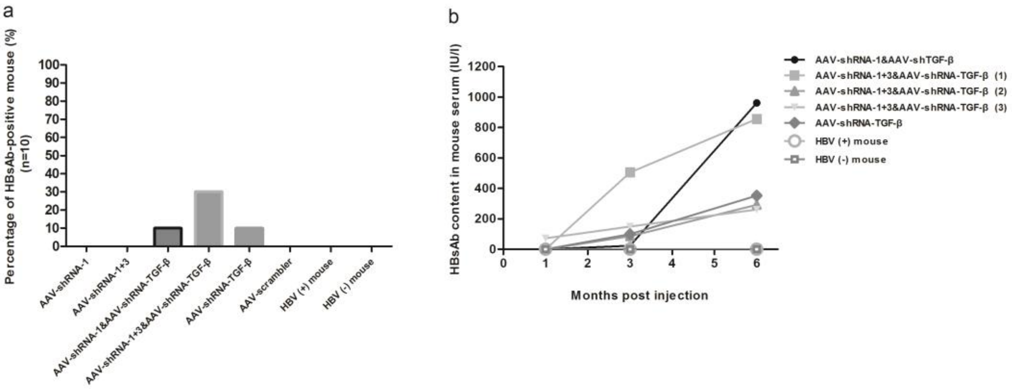

Supplementary Figure S3. AAV-shRNA targeting TGF-β increases the anti-HBsAg seroconversion rate.

(a) Percentage of HBsAb-positive mice in each group receiving different treatments.

(b) Time-course of the HBsAb content change in each HBsAb-positive mouse.

Supplementary Table S1. The sequence of shRNAs for HBV

Oligos	Sequence (5'→3')
shRNA-1	TTCCAGGATCCTCAACAAC
shRNA-2	GTCTGTACAGCATCTTGAG
shRNA-3	TGTCAACGACCGACCTTGA

Supplementary Table S2. The primers for RT-qPCR

Primer	Sequence(5'→3')
RT-q-Col I-f	GACATGTTCAGCTTTGTGGACCC
RT-q-Col I-r	AGGGACCCTTAGGCCATTGTGTA
RT-q-Col III -f	TTTGGCACAGCAGTCCAATGTA
RT-q-Col III -r	GACAGATCCCGAGTCGCAGA
RT-q-TGF-β1-f	CTGCTGACCCCCACTGATAC
RT-q-TGF-β1-r	GTGAGCACTGAAGCGAAAGC
RT-q-α-SMA-f	AGCCAGTCGCCATCAGGAAC
RT-q-α-SMA-r	GGGAGCATCATCACCAGCAA
RT-q-β-actin-f	CGTTGACATCCGTAAAGACC
RT-q-β-actin-r	TAGAGCCACCAATCCACACA
